# Restoring Access in a Thrombosed Hemodialysis Reliable Outflow Graft Using the InThrill Thrombectomy System

**DOI:** 10.7759/cureus.56496

**Published:** 2024-03-19

**Authors:** Rakesh Reddy Devireddy, Osama Qaqi

**Affiliations:** 1 Internal Medicine, Garden City Hospital, Garden City, USA; 2 Interventional Cardiology, Garden City Hospital, Garden City, USA; 3 Interventional Cardiology, Michigan Outpatient Vascular Institute, Dearborn, USA

**Keywords:** arteriovenous graft, vascular access, hemodialysis access, mechanical thrombectomy (mt), arteriovenous access for renal dialysis, vascular access failure, hero graft, dialysis access thrombosis

## Abstract

Arteriovenous grafts (AVGs) provide vascular access for hemodialysis in patients with end-stage renal disease (ESRD). However, vascular access thrombosis often occurs, requiring frequent reinterventions to maintain access patency. This report describes the successful use of the InThrill Thrombectomy System (Irvine, CA: Inari Medical) for macerating and removing thrombus from an occluded and heavily thrombosed AVG. A 47-year-old male was sent to our institute for a thrombosed right upper extremity arteriovenous access with a HeRO graft (South Jordan, UT: Merit Medical). The patient underwent interventions for the thrombosis of the same AVG two weeks prior using an Aspirex catheter (Franklin Lakes, NJ: BD Medical), and again two days prior with a Fogarty balloon catheter (Irvine, CA: Edwards Lifesciences). The patient presented with a recurrent completely occluded AVG. Using the InThrill Thrombectomy System and balloon angioplasty, the stenosis was reduced to less than 20%, resulting in brisk flow. The patient tolerated the procedure well without complication and recovered in the holding area with no acute distress. He was discharged the same day on anticoagulation therapy.

This study highlights the successful use of the InThrill Thrombectomy System for the treatment of thrombosed AVG in a hemodialysis-dependent ESRD patient. The device was easy to use and efficient. Device and procedure times are unparalleled when compared with thrombolytic-based procedures. The patient’s AVG remained patent at a 14-day follow-up.

## Introduction

Hemodialysis through vascular access has been the dominant treatment modality for patients with end-stage renal disease (ESRD). Therefore, maintaining functional dialysis vascular access is crucial for patients on maintenance hemodialysis. Arteriovenous grafts (AVGs) are one of the common vascular accesses used for hemodialysis. AVGs can be functional within days following their creation but are associated with a higher risk of venous stenosis and thrombosis compared to arteriovenous fistulas (AVFs), with more than 50% of AVGs reported to thrombose within one year of creation [1]. Dialysis vascular access thrombosis is the leading cause of AVF and AVG failure [2].

Current percutaneous thrombectomy treatment options frequently use thrombolytics, which are associated with serious complications including acute major bleeding, and/or rely on continuous aspiration, which can result in large volumes of intraprocedural blood loss, and similar to thrombolytics, are ineffective against more organized thrombus [3-5]. An effective, thrombolytic-free approach is needed to safely remove the full spectrum of thrombi.

This case describes the successful use of the InThrill Thrombectomy System (Irvine, CA: Inari Medical), a minimally invasive mechanical thrombectomy device, for macerating and removing thrombus from a totally occluded and heavily thrombosed AVG in an ESRD patient on hemodialysis treatment. This over-the-wire 8-French (F) system consists of the InThrill sheath and the InThrill thrombectomy catheter. The InThrill sheath, equipped with a recapturable, distal-braided funnel and a side port for aspiration, is designed to simplify insertion, repositioning, and thrombus removal. The InThrill catheter features a self-expanding nitinol coring element and a working length of 65 cm for precise positioning and versatility for use in 4-10 mm vessels.

This case was previously presented as a poster at the 2023 Vascular Scientific Sessions organized by the Society of Vascular Medicine on September 8, 2023.

## Case presentation

A 47-year-old male was referred to our institute by his nephrologist for dialysis access evaluation by angiography with possible intervention. His medical history included ESRD, diabetes mellitus, hypertension, hyperlipidemia, and recurrent thrombosis of a right upper extremity AVG with HeRO graft (South Jordan, UT: Merit Medical). Prior to the current intervention, the patient underwent an intervention for the thrombosis of the same AVG two weeks prior using an Aspirex Thrombectomy System (Franklin Lakes, NJ: BD Medical), and again two days prior using a Fogarty balloon catheter (Irvine, CA: Edwards Lifesciences). Clinical examination of the patient showed mild swelling over the AVG and absence of thrill and bruit. At presentation, the patient was not on antiplatelet or anticoagulant medication.

**Figure 1:**
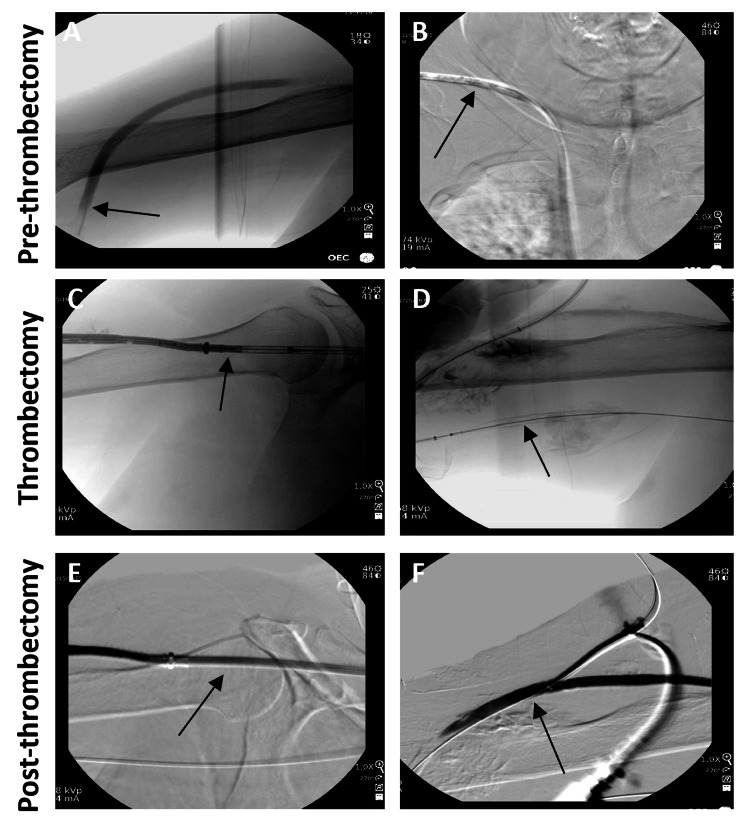
Venography images of the AVG and HeRO graft (South Jordan, UT: Merit Medical) pre-, during, and post-thrombectomy. (A) Thrombosed AVG connected to a HeRO graft. (B) Thrombus in the HeRO graft. (C) AVG to HeRO graft with InThrill catheter (Irvine, CA: Inari Medical) inserted. (D) Brachial artery with InThrill catheter inserted. (E) Restored contrast flow indicating successful thrombectomy. (F) Successful thrombectomy from the brachial artery. AVG: arteriovenous graft

Intravenous conscious sedation was initiated. Using palpation and ultrasound as guidance, percutaneous access of the right brachiocephalic AVG at the area of the anastomosis was obtained. A 6-F sheath was introduced and was flushed with 3000 units of heparin. Selective serial outflow AVG angiography was completed to the superior vena cava. Then, after compressing the distal outflow graft, a selective arterial inflow anastomosis fistulogram was completed (Figure 1, panel A). Next, using an 0.035 cm stiff angled glidewire (Somerset, NJ: Terumo Corp.) and a 5Fr 0.035-seeker support catheter (Franklin Lakes, NJ: Becton, Dickinson), cannulation of the totally thrombosed and occluded right AVG was achieved, and a selective outflow angiography was completed, showing a completely occluded HeRO graft (Figure 1, panel B).

**Figure 2:**
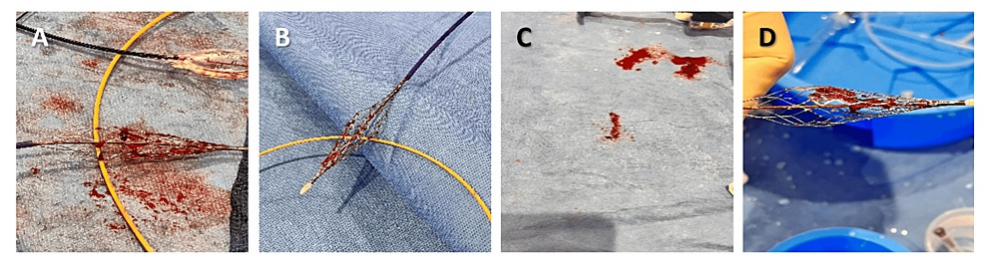
Images (A-D) of the InThrill coring element (Irvine, CA: Inari Medical) and captured thrombus after multiple passes with the device.

The 6-F sheath was then exchanged for an 8-F InThrill sheath with the sidearm flushed with heparinized saline, administering 3000 units of heparin. Thrombectomy of the occluded graft was performed using the InThrill thrombectomy catheter (Figure 1, panels C and D). The catheter was passed multiple times from the outflow through the HeRO graft, removing chronic appearing thrombus (Figure 2, panels A-D). Thrombectomy was repeated until there was no more thrombus to remove (Figure 1, panels E and F).

**Figure 3:**
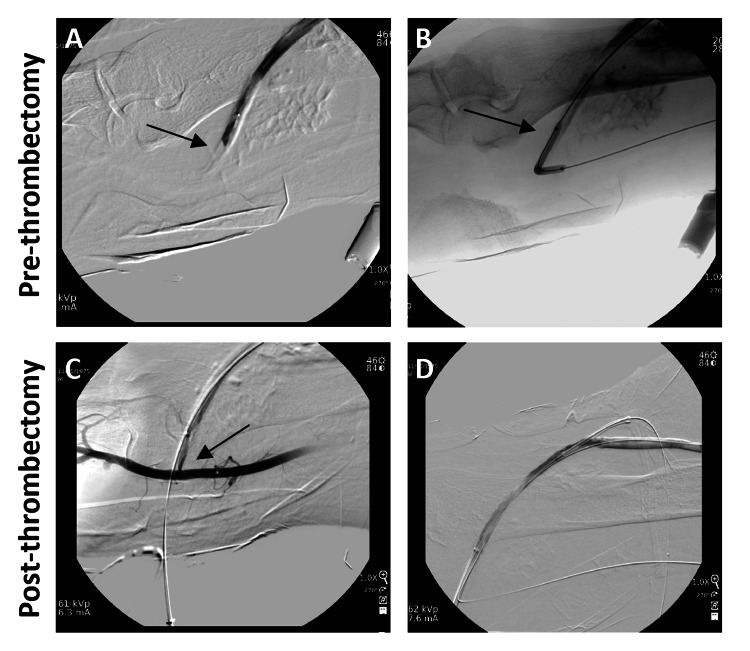
Venography images pre- and post-thrombectomy of the artery-vein anastomosis. (A) Artery-vein anastomosis with thrombus. (B) Angioplasty of the artery-vein anastomosis with ballooning. (C and D) Anastomosis with established flow.

Next a 5.0x100 mm balloon was placed into the AVG and dilated nominally for 3 minutes. The balloon was then deflated, pulled back proximally, and dilated mildly for 3 minutes. This was repeated proximally. The balloon was then deflated and removed intact. Sluggish flow was noted due to the presence of an arterial plug at the anastomosis (Figure 3, panels A and B). Using palpation as guidance and a micropuncture needle, additional percutaneous access was gained approximately 5 cm distal to the anastomosis. A 6-F sheath was introduced and was flushed with heparinized saline. Next using a 035-glidewire and 0.035-seeker support catheter, we were able to cannulate through the arterial anastomosis into the brachial artery (Figure 3, panels C and D). A good bruit and thrill were noted indicating a patent AVG with venography showing less than 20% residual stenosis. Hemostasis was achieved using manual pressure.

The InThrill device time was 30 minutes, with a procedure time of 60 minutes and an estimated blood loss of 50 mL. The patient tolerated the procedure well without complication and recovered in the holding area with no acute distress. He was discharged the same day on rivaroxaban anticoagulant therapy. At a two-week follow-up phone call, the patient reported a patent and functional AVG. The patient was advised to continue follow-up with their nephrologist.

## Discussion

This article highlights the successful use of the InThrill Thrombectomy System for the treatment of a thrombosed AVG in a hemodialysis-dependent ESRD patient. The InThrill system has been successfully used in hemodialysis access thrombosis; however, there are no reported cases where this device has been used for thrombectomy of a HeRO graft for hemodialysis vascular access in an outpatient setting [6,7].

The diagnosis of dialysis access thrombosis is usually made on clinical grounds. The absence of thrill, bruit, or pulse within the vascular access is the key finding on physical examination [5]. Once access thrombosis has been diagnosed and confirmed with ultrasonography (not often required), an intervention should be performed as soon as possible to improve dialysis access survival and avoid the need for a central venous catheter [8]. Mechanical thrombectomy allows for rapid thrombus removal, without the extended dwell times needed with thrombolytic-based therapies.

As with other interventions, thrombectomy is not without risks. Access infection, pulmonary hypertension, recent access creation, severe ipsilateral steal syndrome, and right-to-left shunt are absolute contraindications for endovascular thrombectomy of vascular access thrombosis. Additionally, hyperkalemia (K>6mEq/L), fluid overload, and hemodynamic instability are considered temporary contraindications [1,9]. Furthermore, several complications including arterial embolism, pulmonary embolism, vessel rupture, and peri-access hematoma can occur during dialysis vascular access thrombectomy. Arterial embolism is the most feared complication of thrombectomy with a reported incidence of 0.4-7% [1]. Actions that can reduce the risk of arterial embolism include minimizing manual compression of the vascular access, avoiding flushing of the sheaths after placement, avoiding reflux angiograms, and completely deflating the balloon when attempting to pass the balloon across the arterial anastomosis into the feeding artery. Treatment options for arterial embolism include back bleeding, balloon embolectomy, thrombolytic infusion, and surgical embolectomy [1,9].

The incidence of clinically symptomatic pulmonary embolism following thrombectomy of thrombosed dialysis access is very low [1]. However, dialysis patients have been shown to have more than 12 times the mortality rate due to pulmonary embolism compared to the general population, emphasizing the importance of minimizing cardiac strain and releasing emboli into the central venous system [10]. This is of even greater importance when treating a thrombosed HeRO graft, which connects the brachial artery directly to the right atrium to restore central outflow. This direct path from the artery to the right atrium poses a greater potential risk for pulmonary emboli during thrombectomy and thus underlines the importance of thrombus removal, rather than maceration alone [11]. Importantly, the self-expanding funnel of the InThrill sheath allows for wall apposition to assist with complete thrombus capture and removal with the InThrill coring element, unlike techniques that rely on macerating thrombus with no mechanism for thrombus removal.

## Conclusions

Overall, in this experience, the device was safe, easy to use, and efficient in thrombus removal to restore a thrombosed AVG and maintain patency through 14 days. In our experience, device and procedure times are unparalleled when compared with thrombolytic-based procedures, while avoiding the inherent bleeding risks of thrombolytics. The positive results observed in this report encourage further study of the safety and long-term effectiveness of the device in treating dialysis access thrombosis.
